# To Be Expressive or Not: The Role of Teachers’ Emotions in Students’ Learning

**DOI:** 10.3389/fpsyg.2021.737310

**Published:** 2022-01-17

**Authors:** Yang Wang

**Affiliations:** School of Educational Science, Nanjing Normal University, Nanjing, China

**Keywords:** teacher’s emotions, students’ learning, enhanced-expression, social presence, arousal level, cognitive load

## Abstract

Understanding the role of teachers’ facial expressions in students’ learning is helpful to improve online teaching. Therefore, this study explored the effects of teacher’s facial expressions on students’ learning through analyzing three groups of video lectures. Participants were 78 students enrolled in three groups: one with an enhanced-expression teacher, one with a conventional-expression teacher, and one with the teacher’s audio only. ANOVA was used to explore whether video lectures instructed by the enhanced-expression teacher were better than those instructed by the conventional-expression teacher and the audio-only teacher for facilitating students’ learning, and what is the role of the teacher’s emotions in students’ perceived social presence, arousal level, cognitive load, and learning. The results showed that the video lecture by the enhanced-expression teacher was better than those with the conventional-expression teacher and with the audio-only for facilitating students’ social presence, arousal level, and long-term learning. Interestingly, it was found that the teacher’s emotions could relieve students’ cognitive load. These results explained the inconsistency of existing studies by exploring the mechanism of teachers’ emotions in students’ learning. It also provides teachers with practical guidance for video lecture design.

## Introduction

Video lectures are effective for delivering knowledge and have attracted much attention from practitioners and researchers. During the COVID-19 epidemic, video lecture-based learning has made it possible for students to learn at home without face-to-face interaction, avoiding the risk of virus transmission. However, how to minimize students’ negative emotions, such as isolation, anxiety, and loneliness during online learning is one of the biggest challenges faced by teachers and researchers (Zembylas et al., 2008; Reupert et al., 2009; Regan et al., 2012; Xing et al., 2019). Studies support that teachers’ emotions play a key role in students’ learning. For example, Zembylas et al. (2008) found that teachers’ emotional support is essential to relieve students’ negative emotions. Reupert et al. (2009) examined the importance of teachers’ presence and emotions for students’ online learning. Fanselow (2018) found that teachers’ emotions are associated with students’ attention, memory, and motivation. Wang et al. (2019) supported that teachers’ emotions had a positive effect on students’ satisfaction and learning. Additionally, students’ emotions depend heavily on the teacher’s teaching design (Frenzel et al., 2018; Mainhard et al., 2018). It is how teaching behaviors are interpreted by students that determines students’ learning rather than the learning materials themselves (Marsh and Bailey, 1993). The Emotional Response Theory also supports that the connection between teacher communication and students’ learning is regulated by the students’ responses to the teacher’s emotions (Mottet et al., 2006).

Although it is supported that teachers’ emotions are essential to students’ learning, teachers have not realized the role of their emotions in students’ learning in video lectures. Some teachers tend to demonstrate relatively enhanced emotions, while others prefer to be conventional. Some teachers even feel that their expressions should be serious, as education itself is a serious matter (Wang et al., 2019). Hence, an understanding of the role of teachers’ emotions in students’ learning can provide teachers with practical guidance as to how to behave in video lectures.

The role of emotions in education has recently attracted researchers’ attention, and it is now widely accepted that teachers’ emotions are essential to students’ learning. For example, Neill (1989) found that the teacher’s emotions facilitated students’ learning interest and performance. Similarly, Theonas et al. (2008) supported the belief that teachers’ emotions expressed through facial expressions would improve students’ learning. Wang et al. (2019) analyzed the role of teachers’ facial expressions in students’ learning and found that teachers’ expressions improved students’ learning. Keller and Becker (2020) explored the role of teachers’ emotions in students’ responses and found that teachers’ emotions were related to students’ emotions. Although studies support that teachers’ emotions are essential to students’ learning, there are still some studies which have found that the presence of the teacher had no significant effects on students’ learning (Homer et al., 2008; Kizilcec et al., 2014). That is, the effects of teachers’ presence and emotions on students’ learning are still to be further explored. Exploring the role of teachers’ presence and emotions in students’ perceived social presence, emotions, cognitive load, and learning can improve our understanding of the internal mechanism of teachers’ emotions in students’ learning. It would also be helpful for effective video lecture design.

## Literature Review

### Emotions

Emotions are involved in every aspect of our lives, and they are key elements in education (Lacave et al., 2020). Emotion itself is difficult to define. It is an experience of a subject’s attitude toward an object mediated by the central nervous system (Kleinginna and Kleinginna, 1981). Scherer (2009) proposed that emotions can be defined as cognitive, motivational, and physiological processes. They influence the way people think, behave, and process social information (Forgas and Eich, 2012). Despite the complex structure of emotions, currently emotions are often divided into two categories: positive and negative emotions (Watson et al., 1988). Positive emotions are often associated with optimism, happiness, and confidence, while negative emotions are associated with isolation, anxiety, and tension. It is supported that positive emotions are often associated with a better competence to pick relevant from irrelevant information, resulting in better performance in cognitive skills (Moridis and Economides, 2009). On the other hand, negative emotions are often associated with information analysis and judgment making (Isen et al., 1985). It is also supported that students’ emotions affect their attention, cognition, and social behaviors (Isen et al., 1987; Chen and Sun, 2012). Lacave et al. (2020) also found that students’ emotions are essential to their learning. Although emotions are essential to students’ cognitive process, the internal mechanism of teachers’ emotions in students’ learning is still to be further explored. Kizilcec et al. (2015) supported that most students favor lectures with the teacher’s presence, while some studies have proposed that teachers’ emotions harmed students’ learning (Homer et al., 2008). That is, there is no consensus on the role of teachers’ emotions in students’ learning. Hence, exploring the role of teachers’ emotions in students’ perceived social presence, arousal level, cognitive load, and learning can further explain the mechanism of emotions in students’ learning. It is proposed that facial expressions can reflect a person’s emotions in a natural state (Ekman et al., 1978; Keller and Becker, 2020). Thus, teachers’ and students’ emotions were analyzed through facial expressions in this study.

### The Role of Teacher’s Emotions in Students’ Perceived Social Presence, Emotions, Cognitive Load, and Learning

Although teachers’ emotions are essential to students’ learning (Wang et al., 2019), the specific role of teachers’ emotions in students’ perceived social presence, arousal level, cognitive load, and learning have yet to be explored.

#### The Role of Teachers’ Emotions in Students’ Perceived Social Presence

Social presence refers to the extent to which a student is perceived as a “real person” in communications supported by media (Gunawardena, 1995). It is the extent to which a student authentically engages in learning interaction with others to reduce the distance between them. Social presence is determinant of students’ online learning assessment (Edwards, 2021). Affective expressions, open communication, and group collaboration are the three factors that determine students’ social presence. The affective expressions represent the extent to which participants share their personal details and emotions. Open communication refers to the extent to which group members trust in group communication and share ideas freely. Group coherence refers to the extent to which group members communicate frequently and effectively, and their readiness to influence and be influenced by others (Byrne, 2021). The perceived social presence determines students’ perceived distance during online learning. Additionally, this perceived distance between students and teachers in the online environment is a considerable challenge for online learning (Barratt and Duran, 2021). The community of inquiry theory supports that teachers’ expressions have effects on students’ perceived presence and online learning (Borup et al., 2012). The emotion expressed by the teacher’s expressions in lectures could shorten this distance (Wang et al., 2019). However, Homer et al. (2008) found no significant differences between the social presence of students who learned in the video or no-video conditions. Thus, exploring the role of teachers’ emotions in students’ perceived social presence would be valuable for improving our understanding of online teaching.

#### The Role of Teachers’ Emotions in Students’ Arousal Level

Arousal level is a person’s physiological and psychological awakening state. It is a measure of a person’s perceived energy level, ranging from low (calming) to high (exciting). Arousal has been examined to be associated with cognition, psychology, and learning (Bjalkebring et al., 2015; Hoogerheide et al., 2019). Hence, exploring the effects of a teacher’s emotions expressed by facial expressions could improve our understanding of the effects of teachers’ emotions on students’ learning in video lectures. Arousal levels can be assessed through the change of facial action units (Senechal et al., 2012). A two-dimensional space constructed by Ressel (1980) determined emotions according to two aspects: arousal (inactive-active) and valence (unpleasant-pleasant). The results were validated with facial expression stimuli (Calbi et al., 2017; Schneider et al., 2020).

Teachers’ emotions have been shown to be associated with students’ attention, memory, and motivation (Fanselow, 2018). It has even been found that emotions demonstrate stronger predictive power for students’ learning performance than motivation and cognition (Ruiz et al., 2016; Iskrenovic-Momcilovic, 2018). Robinson (2013) found that teachers’ emotions had a positive effect on students’ satisfaction and learning. Pekrun (2014) also found that students’ perceived emotions during computer-supported learning were essential to their learning performance. Furthermore, students’ emotions greatly depend on the teacher’s emotions (Frenzel et al., 2018; Mainhard et al., 2018). Although it is supported that teachers’ emotions determine students’ learning, the role of teachers’ emotions in students’ arousal level needs to be explored to explain the role of teachers’ emotions in students’ video-lecture-based learning.

#### The Role of Teacher’s Emotions in Students’ Cognitive Load

Intrinsic and extraneous load are two essential aspects of cognitive load (Sweller et al., 2019). Intrinsic cognitive load stems from the difficulty of the learning content, while extraneous cognitive load is generated from the poor design of the learning materials. Since a learner’s cognitive resources in the information processing system are limited, Mayer and Moreno (2003) proposed that the cognitive load from the learning media is the biggest challenge of multimedia-supported education. Homer et al. (2008) proposed that, compared with students in the group without the teacher’s video, those in the group with the teacher’s video experienced a relatively higher cognitive load. Students may feel relaxed when teachers demonstrate positive emotions, while they may feel frustrated when the teacher seems negative. Students may have difficulty when they struggle to understand the teacher’s emotions and the reasons behind those emotions (Schutz and DeCuir, 2002). That is, the teacher’s emotions may improve students’ cognitive load. However, Kizilcec et al. (2015) reported that most students tend to learn from lectures with the teacher’s presence, and supported that the teacher’s presence was essential to students’ video-lecture learning. Wang et al. (2019) also supported that teachers’ emotions have effects on students’ learning and satisfaction. That is, the role of teachers’ emotions in students’ cognitive load is still to be further explored as exploring the role is essential to explain the mechanism of teachers’ emotions in students’ learning, and can provide practical guidance to improve students’ video-lecture learning.

#### The Role of Teachers’ Emotions in Students’ Learning

Social learning theory proposes that learning is a process of observing, analyzing, and imitating others’ emotions, attitudes, and behaviors (Bandura, 1997). This theory considers environmental and cognitive factors as two major elements that influence learning. It is proposed that emotions evoked in the learning can influence the learning process in turn. Robinson (2013) explored the interrelationship of emotion and cognition in online courses, and found that emotions can influence students’ motivation and observation. Kay (2008) analyzed the relationship between emotions and students’ learning and found that positive emotions can improve students’ learning. However, Homer et al. (2008) and Kizilcec et al. (2014) found no significant difference between the learning performance of students who learned through lectures with and without the teacher’s presence. That is, there is no consensus on the role of teachers’ emotions in students’ learning.

### Research Questions

Although studies support that teachers’ emotions are essential and can influence students’ emotions, the internal mechanism of teachers’ emotions in students’ learning is still to be further explored. To fill this gap and to provide practical guidance for video-lecture teaching, the role of teachers’ emotions in students’ perceived social presence, arousal level, cognitive load, and learning were explored in this study with quasi-experimental research.

Specifically, this study aimed to explore the following research questions:

RQ1: What is the role of the teacher’s emotions in students’ perceived social presence?

RQ2: What is the role of the teacher’s emotions in students’ arousal level?

RQ3: What is the role of the teacher’s emotions in students’ cognitive load?

RQ4: What is the role of the teacher’s emotions in students’ learning?

## Materials and Methods

### Study Design

A quasi-experiment was conducted to analyze the role of the teacher’s emotions in students’ perceived social presence, arousal level, cognitive load, and learning. Participants were randomly assigned into three groups and students of the three groups learned with video lectures instructed by a teacher with different levels of emotions. In the first group, students learned with the video lecture instructed by an enhanced-expression teacher; those in the second group learned with the video lecture instructed by the same teacher with conventional expression; and students in the third group learned with the teacher’s audio of the lecture only. Then students’ perceived social presence, emotions, cognitive load, and learning data were collected to analyze the mechanism of the teacher’s emotions in the students’ learning.

### Participants and Materials

In this study, participants were students recruited from a Chinese university through the social network software, QQ. They were normal in terms of their social communication and had no previous learning experience of micro-course design. The fact that the learning process would be recorded was communicated to all participants before the experiment. Participants’ background information including gender, age, and educational level was collected. The experiment schedules were defined during the enrollment. Of the 89 students who registered, 78 were available for the experiment. Participants’ ages ranged from 18 to 24 years old (*M* = 20.4, *SD* = 1.93) with 15 males and 63 females.

Participants were randomly assigned to three learning groups. There were 26 students (5 males and 21 females) in each group. The teacher of the three video lectures was a female teacher with more than 4 years of online teaching experience.

The topic of the three video lectures was micro-course design. The content and teaching design of the three lectures were the same. In the conventional-expression video lecture the teacher was informed to teach in her usual way. In the enhanced-expression video lecture, she was encouraged to express her emotions as clearly as she could. The audio of the audio-only lecture was taken from the enhanced-expression lecture.

### Measurements

#### Perceived Social Presence

To explore the role of the teacher’s emotions in students’ perceived social presence, students’ perceived social presence was investigated after the learning session with a questionnaire adapted from Kim and Biocca (1997). Perceived social presence was assessed using six items (as shown in Table 1). Each question was assessed with a 5-point Likert scale (1 “*totally disagree*,” 5 “*totally agree*”). There was a reverse question in the social presence scale and its score was reversed before the data analysis. Hence, a higher score indicated a higher perceived social presence. The Cronbach’s reliability (alpha) for perceived social presence was 0.89 which is considered acceptable (Nunnally, 1978).

**TABLE 1:** The six-item questionnaire for the measurement of perceived social presence.

Dimension	Definition	Num	Item
Social presence	The sense that another person is “real” and “there” when using a communication medium	1	During the video-lecture learning, I feel like I am in a world constructed by the teacher.
		2	During the learning, I NEVER forgot that I was in the middle of an experiment.
		3	During the learning, my body was in the room, my mind was with the teacher.
		4	The lecture came to me and created a new world for me, and the world suddenly disappeared when the lecture ended.
		5	When the lecture finished, I felt like I came back to the “real world” after a journey.
		6	When the lecture finished, I could not come back to the real world in a short time.

#### Emotions and Arousal Level

Facial expressions analysis based on FaceReader is more reliable compared with traditional investigation and observation (Sun and Chen, 2016). FaceReader was constructed according to Ekman’s basic emotion theory. In FaceReader, emotions and arousal levels are calculated based on movement analysis of 20 facial action units (Ekman et al., 1978). It has been found to be reliable and effective for assessing a person’s emotions (Drozdova, 2014). Given the reliability of FaceReader, it was used in this study to assess the teacher’s and students’ emotions. The three video lectures were imported into FaceReader to explore the teacher’s emotions. Students’ learning processes were recorded with the camera in the computer, and FaceReader was used to analyze the recorded videos to determine the students’ arousal levels.

#### Cognitive Load

To explore the role of the teacher’s emotions in the students’ cognitive load, students’ perceived social presence and cognitive load were investigated after the learning session with a questionnaire adapted from Leppink et al. (2013). Cognitive load was assessed with six questions, including two on intrinsic load, two on extraneous load, and two on germane load (as shown in Table 2). Each question was assessed with a 5-point Likert scale (1-“*totally disagree*,” 5-“*totally agree*”). The Cronbach’s reliability for the cognitive load scale was 0.90. The reliability for each sub-scale (intrinsic load, extraneous load, and germane load) was calculated to be 0.87, 0.91, and 0.85, respectively, which is considered to be acceptable (Nunnally, 1978).

**TABLE 2:** The six-item questionnaire for the measurement of cognitive load.

Dimension	Definition	Num	Item
Cognitive load	Intrinsic load	1	What I just learned is very complex.
		2	The concepts or problems involved in the video lecture are very complex.
	Extraneous load	3	The instructions and/or explanations were very unclear.
		4	The teacher’s expressions in the video lecture were difficult to understand.
	Germane load	5	The video lecture enhanced my understanding of concepts and definitions of micro-courses.
		6	The video lecture enhanced my knowledge of how to design micro-courses.

#### Learning Performance

Before the learning, students’ knowledge of this topic was assessed with a pre-test. After the learning, students’ learning performance was assessed with short-term and long-term recall of the learning content. There were 10 questions on short-term and long-term recall which were different from the pre-test. The scores of the pre- and post-tests were between 0 and 10. Items such as “The advantages of micro-courses” and “How to design micro-courses” were included in the test. The pre- and post-tests were examined by two experts in educational technology to ensure their validity for assessing students’ knowledge of micro-course design.

### Procedure

**FIGURE 1:**
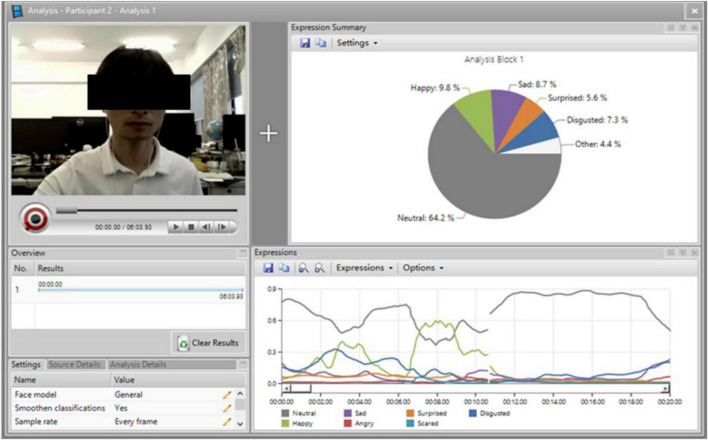
Emotional analysis of a participant.

There were three stages in this experiment. In the first stage, participants were taken into the classroom one by one. Researchers communicated with them to make sure that their social communication skills were normal, and they had not learned micro-course design before. At the same time, they were administered a pretest on micro-courses. In addition, they received training on how to operate the video lecture. In the second stage, they participated in learning. Students were randomly assigned to one of the video lectures. Each participant learned with the video lecture individually. To explore the effect of the teacher’s emotions on the students’ learning, participants’ learning processes were recorded with the laptop camera. The experiment scene is shown in Figure 1. Each student viewed and tried to learn the content. In the third stage, there was a short-term recall posttest on the knowledge after students had finished learning. Their perceived social presence and cognitive load were also investigated. Finally, five participants in each group were interviewed about their learning experience. Two weeks later, a long-term recall posttest was administered to participants through emails. Those who finished the long-term recall posttest were rewarded with cash bonuses. All participants submitted the long-term recall posttest.

## Results

To explore the role of the teacher’s emotions in students’ learning, the two-way ANOVA was used to analyze students’ perceived social presence, arousal level, cognitive load, and learning, using SPSS 19.0. The FaceReader analysis result indicated that there were six facial expressions in the enhanced-expression video lecture, three in the conventional-expression video lecture, and no expression result in the audio-only video lecture.

### The Role of the Teacher’s Emotions in Students’ Perceived Social Presence

**FIGURE 2:**
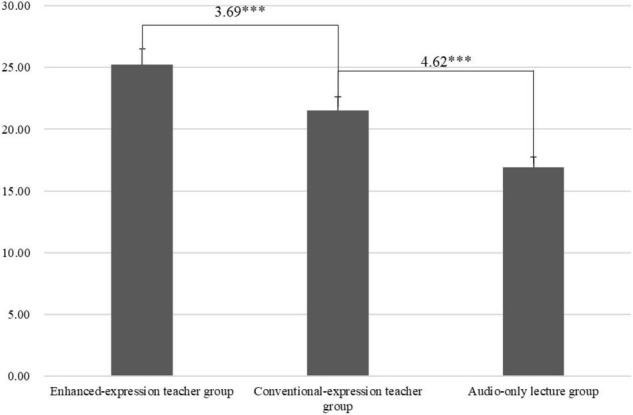
Participants’ perceived social presence in the three groups. The 3.69 and 4.62 represent the Mean difference, ^***^represents that the mean difference is significant at the 0.001 level.

Students’ perceived social presence was analyzed to explore the role of the teacher’s emotions in students’ perceived social presence. The Shapiro-Wilk test supported that we could not refuse the hypothesis that students’ perceived social presence was normally distributed (w_1_ = 0.94, *p*_1_ = 0.11). The two-way ANOVA results indicated that the teacher’s emotions had a significant effect on the students’ perceived social presence [*F*(2, 75) = 53.0, MSE = 112.62, *p* < 0.001]. Specifically, the Bonferroni *post hoc* analysis showed that students’ perceived social presence in the enhanced-expression teacher group was higher than that in the conventional-expression teacher group (*p* < 0.001) and the audio-only lecture group (*p* < 0.001) (as shown in Figure 2). Students in the conventional-expression teacher group demonstrated a higher perceived social presence than students in the audio-only lecture group (*p* < 0.001). RQ1 was thus answered. It was found that students in the enhanced-expression teacher group perceived higher social presence compared with those in the conventional-expression teacher group and the audio-only group. In addition, compared with students in the audio-only group, those in the conventional-expression teacher group perceived higher social presence. That is, the teacher’s presence and emotions improved students’ perceived social presence.

### The Role of the Teacher’s Emotions in Students’ Arousal Level

Students’ arousal level was analyzed to explore the role of the teacher’s emotions in students’ emotions. The Shapiro-Wilk test supported that students’ arousal was normally distributed (w_1_ = 0.89, *p*_1_ = 0.18). The two-way ANOVA results indicated that the teacher’s emotions had a significant effect on students’ arousal level [*F*(2, 75) = 103.1, MSE = 0.682, *p* < 0.001]. Additionally, according to the Bonferroni multiple comparison tests, students’ arousal in the enhanced-expression teacher group was significantly higher than that in the conventional-expression teacher group (*p* < 0.001). Also, students’ arousal in the conventional-expression teacher group was significantly higher than that in the audio-only lecture group (*p* < 0.001). The result answered RQ2. It was found that the students in the enhanced-expression teacher group demonstrated higher arousal level compared with those in the conventional-expression teacher group and in the audio-only lecture group. That is, the teacher’s presence and emotions improved the students’ arousal levels.

### The Role of the Teacher’s Emotions in Students’ Cognitive Load

The Shapiro-Wilk test supported that students’ cognitive load was normally distributed (w_1_ = 0.95, *p*_1_ = 0.19). A significant difference in students’ cognitive load among the three groups was found [*F*(2, 75) = 3.29, MSE = 13.94, *p* = 0.04] through the two-way ANOVA result. Specifically, the Bonferroni multiple comparison tests indicated that students in the enhanced-expression teacher group (*p* = 0.03) and in the audio-only lecture group (*p* = 0.04) manifested significantly lower cognitive load compared with those in the conventional-expression teacher group. No significant difference was found in students’ cognitive load in the enhanced-expression group or in the audio-only lecture group (*p* = 0.71). Additionally, there was no significant difference in students’ intrinsic load and germane load among the three groups. Students in the enhanced-expression teacher group (*p* = 0.04) and audio-only lecture group (*p* = 0.01) reported significantly lower extraneous load compared with those in the conventional-expression teacher group. Table 3 demonstrates the *post hoc* test result of the three groups’ cognitive load. The result answered RQ3. Students in the enhanced-expression teacher group and the audio-only lecture group manifested lower cognitive load compared with those in the conventional-expression teacher group. That is, the teacher’s presence improved students’ cognitive load, but the teacher’s emotions reduced students’ cognitive load.

**TABLE 3:** *Post hoc* test of the three groups’ cognitive load.

Cognitive load	Group (I)	Group (J)	Mean difference (I-J)	Std. error	*p*-value
Intrinsic load	1	2	0.31	0.27	0.57
		3	0.46	0.26	0.15
	2	3	0.15	0.22	0.77
Extraneous load	1	2	–1.42	0.23	0.04*
		3	0.38	0.32	0.32
	2	3	1.46	0.29	0.01*
Germane load	1	2	0.32	0.36	0.48
		3	0.15	0.34	0.76
	2	3	0.12	0.37	0.49

*Group 1 means the “Enhanced-expression teacher group”; group 2 represents the “Conventional-expression teacher group”; group 3 represents the “Audio-only lecture group.”*

*p < 0.05.

### The Role of the Teacher’s Emotions in Students’ Learning

The Shapiro-Wilk test supported that we could not refuse the hypothesis that students’ pre-test performance was normally distributed (w_1_ = 0.96, *p*_1_ = 0.32). Two-way ANOVA supported that no significant difference was found in the pretest knowledge of the three groups [*F*(2, 75) = 0.08, MSE = 0.12, *p* = 0.93]. The Shapiro-Wilk test supported that students’ short-term and mid-term recall were distributed normally (w_1_ = 0.93, *p*_1_ = 0.69). No significant difference was found in the three groups’ short-term recall according to the ANOVA result [*F*(2, 75) = 0.13, MSE = 0.17, *p* = 0.74].

**FIGURE 3:**
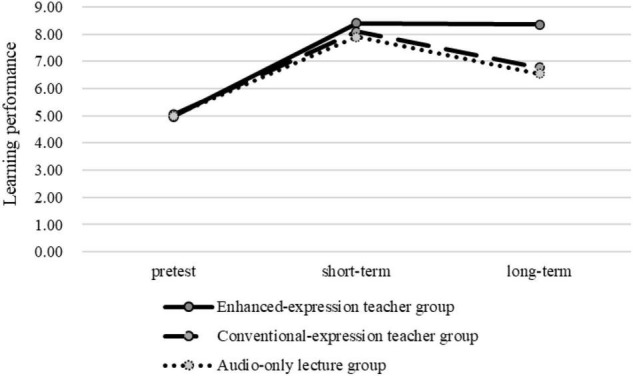
Pretest and posttest outcomes of the three groups.

While a significant difference was found in the long-term recall of the three groups [*F*(2, 75) = 16.42, MSE = 25.17, *p* < 0.001], specifically, students reported higher long-term recall in the enhanced-expression teacher group than those in the conventional-expression teacher group (*p* < 0.001) and the audio-only lecture group (*p* < 0.001) through Bonferroni’s multiple comparison test. No significant difference was found in the long-term recall of the conventional-expression teacher group and the audio-only lecture group (*p* = 0.50). The average scores of the three groups in the pre-test, short-term recall, and long-term recall are reported in Figure 3. RQ4 was therefore answered. That is, compared with students in the conventional-expression teacher group and the audio-only lecture group, those in the enhanced-expression teacher group reported higher long-term recall. It can therefore be stated that the teacher’s presence and emotions improved students’ learning.

## Discussion and Conclusion

The role of a teacher’s emotions in students’ perceived social presence, arousal level, cognitive load, and learning performance was explored. The analysis results supported that the teacher’s emotions facilitated students’ perceived social presence, arousal level, and learning performance. Interestingly, it was found that the teacher’s emotions could relieve students’ cognitive load. Implications of this study are discussed as follows.

### The Role of the Teacher’s Emotions in Students’ Perceived Social Presence

Students in the enhanced-expression teacher group reported a higher social presence than students in the conventional-expression teacher and audio-only groups. Compared with students in the audio-only lecture group, those in the conventional-expression teacher group demonstrated a significantly higher social presence. This finding is consistent with Wang et al. (2019) who found that teachers’ emotions facilitated students’ learning satisfaction. This result can also be explained by the transactional distance theory. This theory supported that students’ perceived communication could influence their perceived distance with the learning community during online learning (Moore and Kearsley, 1996). The distance between students and the teacher is one of the biggest challenges of online learning. Thus, it is essential to shorten the distance between students and the teacher during online learning or video-lecture learning. It was found that the teacher’s enhanced expressions could facilitate students’ perceived social presence, which is essential to shorten the distance between students and the teacher. Hence, it is suggested that teachers could enhance their expressions during video lecture material production.

### The Role of the Teacher’s Emotions in Students’ Arousal Level

It was found that the teacher’s presence and emotions improved students’ arousal level. This finding is supported by Baylor and Kim (2009) who showed that teachers’ emotions expressed through expressions could improve students’ perceptions and learning. It can also be explained by the emotion contagion theory in which the emotional contagion is an automatic, unconscious process of emotional transmission between individuals, an automatic tendency to imitate and synchronize facial expressions and movements, resulting in the production of the same emotion (Liu et al., 2019). The result is also supported by Becker et al. (2014) who found that the teacher’s emotions were determinant of students’ emotions, which could further improve their learning engagement. Thus, it is necessary for teachers to be expressive during video-lecture teaching.

### The Role of the Teacher’s Emotions in Students’ Cognitive Load

The results indicated that the teacher’s presence increased students’ extraneous load, but the teacher’s emotions reduced students’ extraneous load. This result gives teachers practical suggestions for designing effective teaching materials. On one hand, it is consistent with Homer et al. (2008) who proposed that, compared with students in the no-video group, those in the video group experienced a higher cognitive load. On the other hand, it gives teachers practical suggestions to deal with this problem. The teacher’s enhanced expressions during teaching acted as an assisting signal regarding the learning content which could relieve students’ cognitive load. The interview results also supported this finding. Several students proposed that the teacher’s facial expressions during teaching could improve their understanding of the learning content and make it more impressive. This result explained the internal mechanism of why teachers’ enhanced expressions facilitate students’ learning engagement during video lecture learning.

### The Role of the Teacher’s Emotions in Students’ Learning Performance

It was found that the teacher’s presence and emotions had no significant effects on students’ short-term recall. This may have resulted from the low difficulty level of the short-term recall and students’ deep memory of knowledge as they had just finished learning. In contrast, the teacher’s emotions improved the students’ long-term recall. The interview data helped to explain this result. Several students proposed that the teacher’s emotions during teaching acted as an indicator, meaning that the knowledge left a greater impression on them. This result is supported by Wang et al. (2019). Compared with Wang et al. (2019), the current study further explored the role of the teacher’s emotions in students’ social presence and cognitive load, which can explain the internal mechanism of the role of the teacher’s emotions in students’ learning. This result does not mean that the more expressive the teacher is the better, because the cognitive theory proposes that signals that are consistent with the learning content promote student learning (Mayer, 2005). That is, not all rich expressions are useful for students’ learning. It is the emotional support improving students’ understanding of the learning content that is useful for learning. Hence, we can conclude that the teacher’s heightened level of facial expressions during teaching is helpful for students’ content understanding.

This study gives researchers and practitioners practical guidance to improve video-lecture teaching. On one hand, teachers could heighten their facial expressions while teaching to improve students’ learning, as teachers’ improved expression of their emotions facilitates students’ social presence, arousal level, and learning. On the other hand, although it is supported that teacher presence would improve students’ cognitive load, the teacher’s enhanced expressions could relieve students’ cognitive load.

There are some limitations of this study that should be noted. The number of participants in this study was limited, which influences the generalizability of the results. Secondly, the teacher in this study is a teacher who is not so expressive in her natural state. Thus, the result may only apply to such teachers. In the future, more diverse methods can be considered during the analysis to further explore the role of teachers’ emotions in students’ learning.

## Data Availability Statement

The original contributions presented in the study are included in the article/supplementary material, further inquiries can be directed to the corresponding author/s.

## Ethics Statement

The studies involving human participants were reviewed and approved by the Nanjing Normal University. Written informed consent for participation was not required for this study in accordance with the national legislation and the institutional requirements.

## Author Contributions

The author confirms being the sole contributor of this work and has approved it for publication.

## Conflict of Interest

The author declares that the research was conducted in the absence of any commercial or financial relationships that could be construed as a potential conflict of interest.

## Publisher’s Note

All claims expressed in this article are solely those of the authors and do not necessarily represent those of their affiliated organizations, or those of the publisher, the editors and the reviewers. Any product that may be evaluated in this article, or claim that may be made by its manufacturer, is not guaranteed or endorsed by the publisher.
